# *ALU*minating the Path of Atherosclerosis Progression: Chaos Theory Suggests a Role for *Alu* Repeats in the Development of Atherosclerotic Vascular Disease

**DOI:** 10.3390/ijms19061734

**Published:** 2018-06-12

**Authors:** Miguel Hueso, Josep M. Cruzado, Joan Torras, Estanislao Navarro

**Affiliations:** 1 Department of Nephrology, Hospital Universitari Bellvitge, and Bellvitge Research Institute (IDIBELL), L’Hospitalet de Llobregat, 08907 Barcelona, Spain; jmcruzado@bellvitgehospital.cat (J.M.C.); 15268jta@comb.cat (J.T.); 2 Independent Researcher, 08950 Barcelona, Spain; estanis.navarro@gmail.com

**Keywords:** atherosclerosis, *Alu* repeats, noncoding RNAs, miRNA, miRNA sponge, *ANRIL*, chaos theory, Chaos Game Representation, long-range correlations, NF-κB

## Abstract

Atherosclerosis (ATH) and coronary artery disease (CAD) are chronic inflammatory diseases with an important genetic background; they derive from the cumulative effect of multiple common risk alleles, most of which are located in genomic noncoding regions. These complex diseases behave as nonlinear dynamical systems that show a high dependence on their initial conditions; thus, long-term predictions of disease progression are unreliable. One likely possibility is that the nonlinear nature of ATH could be dependent on nonlinear correlations in the structure of the human genome. In this review, we show how chaos theory analysis has highlighted genomic regions that have shared specific structural constraints, which could have a role in ATH progression. These regions were shown to be enriched with repetitive sequences of the *Alu* family, genomic parasites that have colonized the human genome, which show a particular secondary structure and are involved in the regulation of gene expression. Here, we show the impact of *Alu* elements on the mechanisms that regulate gene expression, especially highlighting the molecular mechanisms via which the *Alu* elements alter the inflammatory response. We devote special attention to their relationship with the long noncoding RNA (lncRNA); antisense noncoding RNA in the *INK4* locus (*ANRIL*), a risk factor for ATH; their role as microRNA (miRNA) sponges; and their ability to interfere with the regulatory circuitry of the (nuclear factor kappa B) NF-κB response. We aim to characterize ATH as a nonlinear dynamic system, in which small initial alterations in the expression of a number of repetitive elements are somehow amplified to reach phenotypic significance.

## 1. Atherosclerosis Is a Complex Vascular Disease with Distinctive Traits of Nonlinear Behavior

Atherosclerosis (ATH) is a chronic inflammatory vascular disease that is characterized by the interactions and feedback mechanisms involving lipids, cells, and various molecules and genetic factors [1]. Many of these interactions are nonlinear, and are not proportional to the concentration or density of the stimulus, as they may undergo sudden switches in response to small changes in stimuli. For example, the rate at which the oxidized low-density lipoproteins (oxLDLs) are removed by macrophages is limited by the number of available receptors in the cell membrane [2], and by the complex networks of regulatory and environmental factors [3]. Thus, a systems biology approach through the integration of data from large-scale measurements, such as transcriptomics, proteomics, and genomics, might contribute to the unraveling of the regulatory networks underlying the responses of a wide variety of cellular systems [3]. Mathematical and computational models of such systems show that these effects are not random, but instead, they are qualitatively predictable [4].

Recently, a computational model for plaque development was proposed. This model included 32 immunological parameters that were related to the action of monocytes and macrophages, foam cells, macrophage chemoattractants, endothelium-stimulating cytokines, modified low-density lipoproteins, and high-density lipoproteins (HDLs), as well as the timing of changes in the rate of HDL influx [2]. This model assumed that an injured endothelium would be sufficient for the development of plaques, highlighting the immunological processes that occurred within the arterial walls. Although the model only affords qualitative insight, because of the difficulty in obtaining valid values for all of the required parameters for a quantitative model, it suggests that the nonlinear behavior that is exhibited in the HDL dynamics may lead to sudden changes in plaque behavior [2]. This nonlinear system occurs after periods of growth and equilibrium, in which the plaque environment is altered either because of changes in the composition and function of HDLs with age [5], or to decrease the LDL influx following successful treatment with statins [2].

Some studies have suggested that plaque morphology and biomechanical stress should also be considered as major determinants of plaque vulnerability [6]. Thus, some computational models of blood flow–plaque interactions included data from high-resolution magnetic resonance imaging (MRI), allowing the noninvasive characterization of the plaque geometry. These data also suggested that substantial variations in stress or strain in the plaque under pulsating pressures may lead to plaque rupture [6]. On the other hand, recognition of the main roles of inflammation and immunity in the initiation and progression of ATH has led to the development of mathematical models based on reaction–diffusion equations [7]. Some mathematical models describing the early stage of ATH were developed in an effort to study the recruitment of immune cells from the blood flow via inflammatory cytokines, demonstrating that the chronic inflammatory reaction was developed akin to the propagation of a traveling wave [7]. However, these simplified mathematical models only studied particular aspects of the complex process that was giving rise to ATH, and a comprehensive mathematical model explaining the entirety of the process remains elusive.

Vascular dynamics is also a nonlinear system, showing the time-dependent functional changes that are critically dependent on interactions with various physical forces. These can be described as either extra-tissular (blood flow oscillations, arterial pressure, etc.) or intra-tissular (calcifications, tissue thickness, presence of white cell infiltrates, etc.); however, all of the cases demonstrate an unpredictable evolution as a result of the appearance of minor interferences, with the potential of producing unforeseen outcomes [8]. In this sense, the vascular dynamics can be considered as a “chaotic” system, whose evolution demonstrates a very sensitive dependence on its initial conditions, and follows an uncertain, nonlinear progression [3,8,9]. There are a number of reports supporting this hypothesis. As an example, Bruschke et al. showed that the ATH progression in coronary arteries was a highly unpredictable process that followed a nonlinear course [10], while other studies described the arteries as exhibiting a nonlinear, elastic behavior [11]. Furthermore, carotid arteries exhibited nonlinear variations of circumferential stress and tangent elastic moduli, within the normal pressure range [12], and the evolution of the buckling pressures of arteries under pulsatile pressure conditions was accurately described, using a nonlinear model of elasticity [13]. Lastly, nonlinear models were also used to study the effects of luminal stenosis (and plaque morphology) on plaque stability [14], as well as the interactions between the elastic layer (extracellular matrix (ECM) cap) and the rigid, calcified cells [15].

The majority of the above mentioned studies achieved the characterization of the chaotic behavior of ATH through the unique incorporation of blood flow physics, and its role in the onset and progression of the disease. Nevertheless, there were hints that the nonlinear progression of ATH could also be derived from specific features in regions of the genome encoding the tissues that were involved in ATH, although it is difficult to envision how differential dynamics could be generated in a structure that is as homogeneous and tightly controlled as the mammalian nucleus. In this regard, Xiao et al., through the implementation of a nonlinear prediction method, highlighted a subset of genomic sequences with novel deterministic structures, and nonlinear correlations that were essentially different to those of the exonic and intronic sequences (see Reference [16], and Section 2 for a more in-depth discussion on this topic). These sequences corresponded to members of the *Alu* family of repeated elements, which consisted of short DNA sequences [17] that were involved in the regulation of gene expression [18]. The *Alu* elements were found to be pervasively transcribed in a number of physiological and clinical conditions [19,20,21], and were associated with various human diseases and genetic disorders [22,23].

The nonlinear structural features of *Alu* elements make them worth studying, in an effort to characterize their potential involvement in the generation of a chaotic progression during ATH development. In this review, we describe the possible relationship between the *Alu* repeats and the onset and evolution of ATH, specifically focusing on the unique structural and/or functional features of *Alu* elements, which could contribute to the nonlinear progression of ATH. The three features that were of the most interest were (i) the potential for the *Alu* transcripts to act as microRNA (miRNA) sponges, and hence, impact the general levels of messenger RNA (mRNA) expression; (ii) their ability to generate new regulatory networks via retrotransposition to the gene regulatory sites; and (iii) their disruptive effect on the function of the cardiovascular disease (CVD)-associated long noncoding RNA (lncRNA), antisense noncoding RNA in the *INK4* locus (*ANRIL*), which was a strong risk factor that was associated with ATH.

## 2. Multifractal and Chaos-Theory Analysis of the Human Genome Highlights the Involvement of *Alu* Elements in the Development of Complex, Nonlinear Human Diseases

In Section 1, we drew attention to the various ATH-related physical features, in which disease progression was not directly proportional to the intensity of the physical insults, suggesting that such factors could possibly play a role in the nonlinear nature of ATH. However, we can just as easily hypothesize that ATH evolution is dependent on the nonlinear correlations in the structure of the human genome. In this section, we review a number of mathematical tools and methods (Chaos Game Representation, multifractal analysis, and the detection of long-range correlations), which were applied to the study of the human genome, and gives rise to a role for repetitive elements in the establishment of the nonlinear statistical properties of human genomic DNA.

### 2.1. Chaos Theory Provides Tools for the Analysis of Global Genomic Signatures

The human genome is one of the most intricate molecular machines known to man, and a wide range of approaches are used to study and analyze its complexity. Chaos theory and the Chaos Game Representation (CGR) are two mathematical tools that are often used to characterize highly complex systems. CGR, a combined implementation of chaos theory and chaotic dynamics, is an algorithm that is used for the graphical representation of DNA sequences [24]. The CGR is presented as a scatter plot, in which each point of the plot corresponds to each base in the sequence, thus producing a complex picture of the DNA sequence, in which the local and global patterns of the sequential structure can be defined [24]. In this sense, the CGR represents the statistical properties of the base frequencies as intrinsic properties of the DNA sequence itself [24]. The main interest in the CGR plots and their development [25] stems from their ability to reduce complex DNA sequences into simple visual patterns, facilitating comparative studies of genomic signatures, as well as the analysis of characteristic sequence motifs [26]. In this way, the CGR plots were used to determine not only the degree of variability within and between genomes [27], but also to screen two complete genomes for the presence of mismatches, insertions, or deletions [28,29].

Images that are produced by the CGR can be further broken down through methods that are derived from fractal geometry (multifractal analysis) [24,27]. This approach is especially suited for the analysis of very long strings of information, as it relies on the determination of intrinsic patterns, and has proven useful in revealing the complex motifs in sequences [30]. Pioneering work by Yu et al. has shown that genomic DNA sequences that are isolated from various microorganisms were not random sequences, but contrarily, exhibited strong long-range correlations [31] that were characterizable through multifractal analysis [30]. Later on, the use of multifractal methods was established for the analysis of the human genome [32,33]. Since then, multifractal analysis was used to differentiate between coding and noncoding DNA sequences in humans [34] and bacteria [35], to predict human promoter regions [36], to characterize complete genomes in *C. elegans* and humans [37,38], to perform fast comparisons of the microbial genomes among them [39], to distinguish among isolates of *M. tuberculosis* [40], and even to study the high order of the chromatin structure [38].

CGR and multifractal analysis were also applied to the analysis of protein sequences. Specifically, Yu et al. studied a large number of protein sequences that were derived from corresponding complete genomes, and demonstrated that these protein sequences were, in fact, not completely random in nature [41]. Further developments allowed for the prediction of novel structures of G-protein-coupled receptors (GPCRs) from amino acid sequences, despite the poor degree of homology among them [42], as well as the construction of phylogenetic trees for bacteria, through the use of protein sequences from complete genomes and CGR-based modeling [41].

### 2.2. Mathematical Analysis of the Human Genome Highlights Features of Nonlinear Correlations in the Alu Family of Genomic Elements

The first report of nonlinear correlations in the human genome was put forward by Xiao et al., who used a chaos theory-derived nonlinear prediction method to differentiate between “random” and “non-random” (deterministic) DNA sequences [16]. In their analysis, the authors studied the β-globin locus, which encodes six globin genes (along with their exons and introns), and is enriched with a family of repeated nuclear sequences, named *Alu* repeats (see Section 3). The authors demonstrated that the exonic and intronic sequences in the β-globin locus did not show any significant deviation from a random nature, while the sequences harboring these *Alu* repeated nuclear elements presented nonlinear (deterministic) structures, likely because of a dimeric structure [16]. This intriguing result was eventually confirmed through further work by Moreno et al., who reported that the human genome displayed multifractal behavior, rich in highly polymorphic sequences that were organized into a wide range of combinations [43]. Indeed, this multifractal structure was also seen to be strongly dependent on the presence of *Alu* elements, and more specifically, on the content of *Alu*-S, the oldest and most abundant of the *Alu* family [43].

Another tool that has been used to determine nonlinear statistical properties of DNA sequences is called the “DNA walk”. This analytical method provides a quantitative measure of nucleotide correlation across large distances along the DNA sequence, and has revealed the existence of long-range power–law correlations, which are found exclusively in noncoding regions [44]. This concept was later used to distinguish these regions from protein-coding regions [45]. Although conceptually complex, DNA walks have been interpreted through a multi-level approach. Firstly, the application of DNA walk algorithms to a long DNA sequence (from a chromosome or an entire genome) generates a number of DNA domains in varying sizes, with a range of nucleotide concentrations. Secondly, the long-range correlations identify distant domains with a certain degree of homology. Lastly, the power–law correlation indicates whether or not these distances have followed a nonlinear distribution at the level of either the chromosome or the genome [46]. On this basis, the genomic properties could be partially explained by the presence of clustered repetitive elements, as they would feature in the nuclei of the identified homologous sequences in different DNA domains. Indeed, this seemed to be the case as in a recent body of work, where Sellis et al. showed the existence of power–laws in the size distribution of the lengths separating the consecutive repeats of most of the *Alu* (and long interspersed nuclear element (LINE)) elements in human chromosomes [47]. Despite this, other authors considered that the *Alu* repeats only contributed weakly to these long-range correlations [48]. Surely, these differences in opinion could be explained by the use of different methods and different DNA sequences [49].

**Figure 1:**
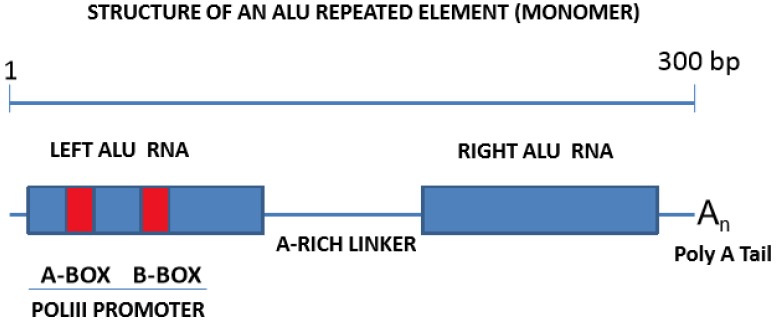
Structural features of *Alu* repeated sequences. Shown here are the distinctive elements forming an *Alu* monomer. The two arms are linked by an A-rich sequence (the bipartite A,B boxes), capped by an RNA polymerase III (Pol III) promoter and a poly-A tail. Graphic not drawn to scale.

The nonlinear correlations discussed above highlighted the *Alu* repeats as a factor contributing to the nonlinear properties of the DNA sequences. Therefore, *Alu* repeats could be considered as genomic parasites, which repeatedly colonized the human genome. Consequently, these events are highly heterogeneous, as a result of the sequence divergence that is dependent on the time at which they were retrotransposed into the genome [17]. The work by Xiao et al. also reflected the distinctive biological functions of *Alu* repeats, on the basis of their dimeric structures, which is reflected in their three-dimensional folding [16]. This dimerization is rather complex as it is formed via two independent 7SL RNA-like folding units (components of the signal recognition particle (SRP)), as well as an inter-domain subunit between the two *Alu* arms [50,51] (Figure 1). In the following sections, we address a number of mechanisms through which the highly heterogeneous *Alu* elements may impact the ATH progression, focusing on the mechanisms through which these *Alu* repeats may affect the gene expression. We also distinguish between the involvement of *Alu*-RNAs and genome-embedded *Alu* elements in these mechanisms.

## 3. The Family of *Alu* Repeated Elements and Their Impact on the Mechanisms Regulating Gene Expression

### 3.1. The Human Genome Is Mostly Composed of Transcribed, Non-Protein-Coding (ncRNA) Genes

The sequencing of various transcriptomes has allowed for the identification of a plethora of noncoding RNAs (ncRNAs), corresponding to genomic regions that were previously referred to as “junk DNA” [52]. According to the latest human GENCODE release (version 28, November 2017), the human genome is composed of fewer than 20,000 protein-coding genes (i.e., a mere 2% of the total genome’s length) [53], in addition to more than 40,000 transcriptional units for previously unclassified non-protein-coding RNAs and pseudogenes (www.gencodegenes.org/stats/current.html). These ncRNAs can be loosely classified into three basic categories, which have been listed below.

(i) Housekeeping RNA, namely, rRNA (ribosomal RNA), tRNA (transfer RNA), snoRNA (small nucleolar RNA), snRNA (small nuclear RNA), Y RNA, SRP RNA (single recognition particle RNA), and 7SK RNA [54].

(ii) Long noncoding RNA (lncRNA, greater than 200 nt), which can be further divided into intronic long intergenic ncRNAs (lincRNA), antisense transcripts from coding regions (antisense transcripts from coding regions (asRNA), which do not encode proteins), circular ncRNA (circRNA) [55], and LINEs (long interspersed nuclear elements) [56], etc.

(iii) Short ncRNA (smaller than 200–300 nt), including microRNA (miRNA) [57], Piwi-interacting RNA (piRNA) [58], and retrotransposon-derived ncRNA, such as short interspersed nuclear elements (SINEs) [59]. Retrotransposon-derived repetitive sequences account for over 50% of the human genome [60].

### 3.2. Alu Repeats: A Family of Highly Succesful Genomic Invaders

The most abundant group of genes encoding ncRNAs are the *Alu* family, a member of the SINE family. *Alu* repeats are established as significantly responsible for the regulation of gene expression and the maintenance of genomic integrity. Considering that *Alu* elements are usually found nearby the gene-rich regions, these repeated elements were shown to impact the regulation of gene expression on both a transcriptional and post-transcriptional level, through various mechanisms [61]. The *Alu* elements are over 300 bp long, and are dimeric retrotransposons composed of two arms, separated by an A-rich linker (Figure 1). *Alu* repeats can be considered as highly successful genomic parasites, which have colonized the human genome, through multiple cycles of retrotranscription (RNA to complementary DNA, cDNA), insertion (cDNA into genomic DNA), and transcription (DNA to RNA), to the extent that approximately one million copies are currently identifiable in the human genome [62]. This means that over 10% of the human genome is composed of *Alu* repeats, which are especially present in gene-rich regions, and that circa 30% of human genes harbor some copy of an *Alu* element [63]. Based on their evolutionary history, *Alu* elements are classified into 12 subfamilies [64], from which only one is currently deemed transpositionally active, while the remainder are inactive, mainly because of 5′ truncation, but also because of sequence degeneration [65]. *Alu* elements are non-autonomous, meaning their reverse transcription and integration into the genome requires the protein machinery of other autonomous retrotransposons, such as LINEs (long interspersed nuclear elements). However, under normal conditions, LINEs are also repressed in the human genome, mostly through promoter methylation [66,67], thus indirectly contributing to the silencing of *Alu* elements. Finally, *Alu* elements have the potential for also modifying the maturation process of mRNAs. On this note, many *Alu* elements were detected in intronic regions [68], where they could provide new signals, resulting in alternative mRNA splicings or even exon skipping [69], or where they can be incorporated into mature mRNAs as “bona fide” exons, either on their own (*Alu* exonization), or after retaining part of the neighboring intron (*Alu*-dependent intronic retention) [70].

### 3.3. Mature Alu-RNAs Include Free Alu Elements Transcribed by RNA Polymerase III, or mRNA-Embedded Alu Elements Transcribed by RNA Polymerase II

Genomic *Alu* repeats include a bipartite polymerase III (Pol III) internal promoter at the 5′ end of the left arm, and a short poly-A tail at the 3′ end of the right arm [18] (Figure 1). This allows their transcription by RNA Pol III through this internal promoter, usually in response to cellular stress [71,72,73]. This process creates “free” *Alu* elements in the form of individual *Alu*-RNA sequences, or alternatively, concatemers of individual *Alu*-RNAs of a yet defined function, which have been detected and cloned in cancer cells [21]. Furthermore, a number of “embedded” *Alu* elements were also detected in mature mRNAs, usually in their 5′ or 3′ untranslated regions (UTRs) [18], with their levels subject to regulation, based on reports of downregulation in cancer cells [74].

**Figure 2:**
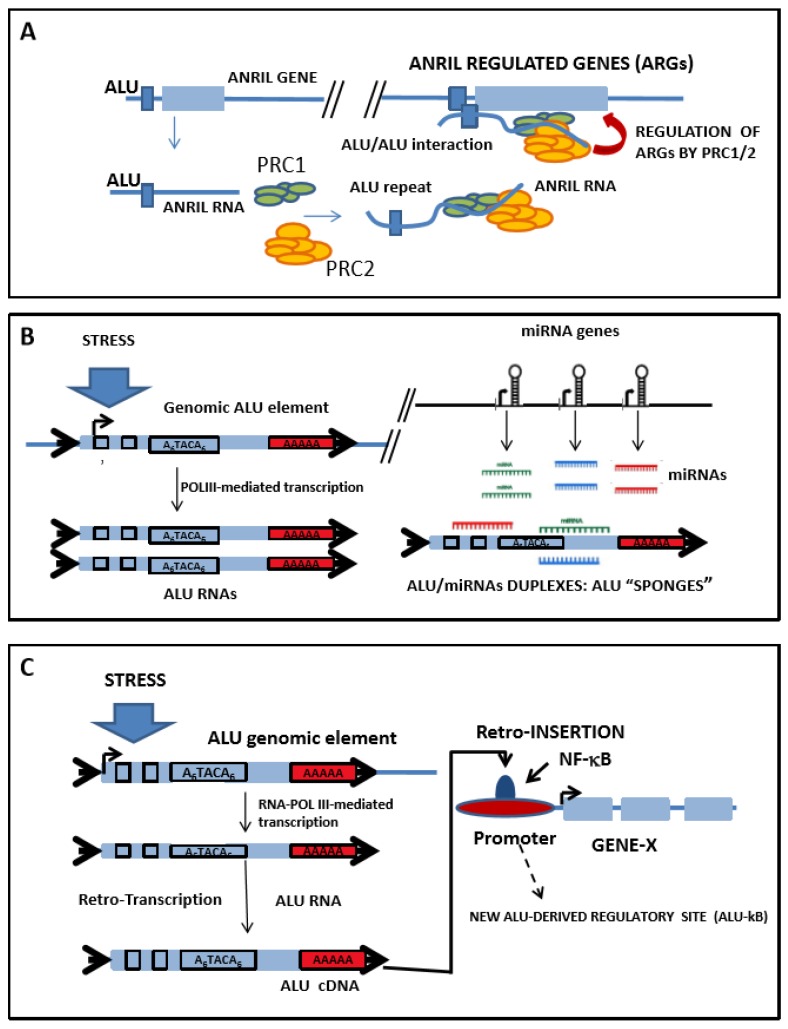
Possible mechanisms through which the *Alu* repeated sequences impact Atherosclerosis (ATH) progression. (**A**) *Alu* elements regulate the function of antisense noncoding RNA in the *INK4* locus (*ANRIL*), a risk factor for atherosclerosis and cardiovascular disease (CVD). *ANRIL* RNA, which harbors *Alu* elements, is transcribed, recruiting polycomb repressive complexes 1 and 2 (PCR1/2), and interacting with other genes via an “*Alu*–*Alu*” or “*Alu*–alternative site” direct interaction, thus facilitating the regulation of their expression through PRC1/2. Taken from Reference [77]. Graphic not drawn to scale. (**B**). *Alu*-RNAs could behave as microRNA (miRNA) sponges, creating complex regulatory networks that are altered in diseases. Shown here are the main elements implicated in the *Alu*/miRNA regulatory loop, namely: *Alu* genes, free *Alu*-RNAs, miRNA genes, and miRNAs. The postulated *Alu*–miRNA interaction does not consider the folding of *Alu* elements, nor does it consider the existence of *Alu* miRNA-binding proteins that could impact the interaction. STRESS stands for any stimulus that upregulates the transcription of free *Alu* elements, such as glucocorticoids [78], human immunodeficiency virus (HIV) infection [79], adenovirus type 5 [80] or type 2 infections [71], herpes simplex virus infection [81], or heat shock [73]. Graphic not drawn to scale. (**C**). Several *Alu* elements are binding sites for transcription factors, such as nuclear factor kappa B (NF-κB), and may impact the gene expression of the inflammatory response. Retrogressed *Alu* elements can function as NF-κB binding sites, thus expanding the set of genes co-regulated by NF-κB in the inflammatory response (see main text for details). Shown here are the main elements implicated in the *Alu*/NF-κB regulatory loop, namely: *Alu* genes, free *Alu*-RNAs, *Alu* complementary DNAs (cDNAs), and their retrogression to gene regulatory regions. STRESS is defined as in (**B**). Dashed arrow shows a new *Alu*-derived regulatory site (*Alu*-κB). Graphic not drawn to scale.

Interestingly, these free *Alu* elements have the potential for impacting the mRNA synthesis, as they were shown to repress RNA Pol II-mediated transcription through the binding of the Pol II initiation complex [18,75]. Furthermore, these free *Alu* elements were considered as probable miRNA targets, likely acting as “miRNA sponges” (Figure 2) [76], and were involved in the regulation of circRNA function circRNA [63] and mRNA stability [17]. In summary, because of their high copy number, their internal promoters, and their embedment into mature transcripts, *Alu* elements are able to impact most of the mechanisms regulating the RNA expression.

### 3.4. Genomic Alu Elements Are Involved in Transcriptional Regulation and Have an Impact on Human Disease

As previously stated, roughly one million *Alu* elements have colonized the human genome via retrotransposition, mostly to gene-rich regions. This invasive process not only had a major impact on the structure of the human genome in normal conditions (health), but also reshaped the genomic landscape for diseases. The first consideration involves the benefits or requirements of an “open” accessible chromatin structure, which the retrotransposition process requires, resulting in retrogressed elements tending to concentrate in regulatory or gene-rich regions. On this topic, a recent work by Gu et al. used technologies based on chromosome conformation capture to demonstrate that the density of *Alu* elements correlated strongly and positively with those of the functional DNA elements, such as enhancers and promoters [82]. Furthermore, there is also functional evidence of the integration of *Alu* elements into human genomic regulatory regions. Without delving into the details, a T-cell-specific enhancer containing an *Alu* element was located in the final intron of the human cluster of differentiation 8 (*CD8*) alpha gene [83], while other nearby repetitive *Alu* elements were able to form a cruciform structure, regulating the function of the *CD8* alpha enhancer [84]. Additionally, the human growth hormone (*HGH*) gene was shown to contain a functional silencing element within an *Alu* repeat in its 3′-flanking region [85]. This close involvement of the *Alu* elements with regulatory regions led several authors to propose that retrogressed *Alu* repeats could form the foundation for new functional sites, such as the Pol II transcription factor binding sites, which would contribute to the generation of new regulatory networks [76], cryptic/alternative splice sites [86], or nuclear receptor binding sites [87]. Therefore, *Alu* elements could be considered as a large reservoir of potential regulatory functions, contributing to the evolution of mechanisms regulating gene expression [69], or even to the creation of novel functional genes [88].

On the other hand, *Alu* elements were also found to be related to the onset of a number of human diseases [22,70] via different mechanisms. These included genetic deletions and duplications [89], insertional mutagenesis [90], or the alteration of methylation patterns in DNA [91]. Correspondingly, genomic regions that were highly enriched with *Alu* elements were considered as intrinsically unstable, since they were targeted by homologous recombination machinery, because of the high homology among the *Alu* sequences [92]. For example, a retroinserted *Alu* element was shown to be the root of neurofibromatosis 1 (NF1), via the inactivation of a downstream exon during splicing, consequently shifting the reading frame of the *NF1* gene [93]. Additionally, a deletion that occurred between two *Alu* repetitive sequences in the same orientation, was shown to inactivate the low-density lipoprotein (*LDL*) receptor gene in Korean patients suffering familial hypercholesterolemia (FH) [94]. Finally, *Alu*-mediated recombinations (leading to exon skipping) were implicated in the origin of Hunter disease [95,96].

## 4. *Anril*: A Long Noncoding RNA Harboring a Risk Factor for Atherosclerosis

Coronary artery disease (CAD) has a heritable trait [97], that is associated with a number of genetic variants [98], and up to 43.5% of the variation in the level of coronary artery calcification (CAC) is attributable to genetic factors [99]. Various genome-wide association studies (GWAS) have identified a strong association between a risk of CAD and a large (58 kbp) intergenic locus at chromosome 9p21 [100] in multiple human populations, including South Koreans [101], Italians [102], Japanese and Koreans [103], American Caucasians [104], Chinese Hans [105], North Indians [106], and Americans of African ancestry [107], among others, while a number of recent meta-analyses have confirmed this association [108,109,110,111]. This region of the chromosome includes several single-nucleotide polymorphisms (SNPs), featuring a tight linkage disequilibrium, disrupting predicted transcription-factor binding sites involved in key physiological processes [112,113]. On a genomic level, the 9p21 risk locus is a protein-coding gene-free region, encoding a long noncoding RNA (lncRNA) called *ANRIL/CDKN2B-AS1* (antisense noncoding RNA in the *INK4* locus, or *CDKN2B* antisense 1), which was identified as a genetic factor associated with cardiovascular morbidity and mortality [114], also correlating with ATH severity [77]. More recently, a targeted deletion of the 9p21 locus was reported, which led to a less stable phenotype in the artery [115], a dependence of diastolic blood pressure and CAC on genetic variation within the *ANRIL* locus [116], and the overexpression of *ANRIL* in ATH-arteries when compared with non-ATH counterparts [117].

In the 9p21 region, *ANRIL* is found relatively far away (over 100 kbp) from the cyclin-dependent kinase inhibitor gene cluster *p15/CDKN2B-p16/CDKN2A-p14/ARF*, despite the first *ANRIL* exon being located adjacent to the *p14/ARF* promoter, overlapping two *p15/CDKN2B* exons [118]. To date, any other transcript remains undetected in this genomic region. Since long ncRNAs are implicated in the regulation of most mechanisms of gene expression, whether transcriptional or post-transcriptional (translational), as well as in the control of mRNA stability, pre-miRNA processing, and chromatin structure [119], the many mechanisms via which *ANRIL* can exert are expectedly diverse [120]. Correspondingly, *ANRIL* reportedly behaves as a miRNA sponge in various diseases, through the targeting of miR-199a [121], miR-125a [122], miR-186 [123], and miR-323 [124], among others. Furthermore, *ANRIL* was also described as a regulator of various signaling pathways, including the ataxia telangiectasia mutated (ATM)/E2F1 pathway [125], the vascular endothelial growth factor (VEGF) pathway [126], and the nuclear factor kappa B (NF-κB) pathway [127]. *ANRIL* was also found to regulate the cell cycle by interfering with the expression of the *p15/CDKN2B-p16/CDKN2A-p14/ARF* locus. *ANRIL* overexpression was also correlated with the downregulation of *p16(INK4a)* [128] and *p15(INK4b)* [129], and was shown to upregulate a number of genes that were involved in proliferation, adhesion, and apoptosis in monocytes [77]. Furthermore, the depletion and mutagenesis of *ANRIL* reversed the trans-regulation of these genes, and normalized the cellular functions [77].

On the other hand, a number of *ANRIL* splicing isoforms were described [77], whose expression, which included exons proximal to the *INK4/ARF* locus, was correlated with an increased risk of atherosclerotic vascular disease (ASVD) [130,131]. *ANRIL* risk alleles were also associated with inflammatory response, as they were shown to disrupt a binding site for the signal transducer and activator of transcription 1 (STAT1) protein, which was responsible for the mediation of the transcriptional response to the gamma interferon (γ-IFN) [132]. Furthermore, it was also suggested that SNPs in risk alleles could alter the profile of the *ANRIL* isoforms at the splicing level, or by generating circular forms of *ANRIL*, which would impact the expression of the neighbouring *p15/CDKN2B-p16/CDKN2A-p14/ARF* locus [133]. Correspondingly, our group reported that the *ANRIL* SNP rs10757278 (GG) doubled the risk of major adverse cardiovascular events (MACEs) in patients with chronic kidney disease (CKD) who were on hemodialysis, via an unknown mechanism [134].

Finally, a recent report revealed an unsuspected functional relationship among *ANRIL* and the members of the *Alu* family of repeated sequences, whereby a new regulatory tier was added to the *ANRIL* activity by *Alu* elements, which impacted the cells’ ability to adhere and proliferate, and facilitated the ATH progression (Figure 2) [77].

## 5. *Alu* Elements May Play Multiple Roles in the Progression of Atherosclerosis

*Alu* repeated elements are connected with ATH progression in a variety of ways, and in this section, we review the most significant of these. From *Alu*-RNAs (whether free or embedded) to *Alu* genomic elements (with various localizations), the *Alu* repeated elements potentially impact a wide range of mechanisms, ensuring the accuracy of gene expression, which can be altered in diseases. Focusing on ATH, we discuss the relationships between the *Alu* elements and two other families of ncRNAs, with a known involvement in ATH progression, namely, a group of small microRNAs (miRNAs), and the lncRNA *ANRIL*, with specific alleles that have been acknowledged as risk factors for CAD (see Section 4).

miRNAs are a class of short ncRNAs (20–22 nucleotides long) that regulate the stability of most of the coding transcripts through the binding of the 3′ UTR of target mRNAs; however, this interaction is highly complex and it is not completely understood [135]. A number of experimental models have highlighted a direct link between the altered miRNA expression and the onset and progression of ATH [135,136,137]. Moreover, miR-21, miR-126, and miR-155 were all characterized as regulators of vessel remodeling [138], while miR-21 and miR-155 were found to regulate foam-cell formation [135,139], as well as miR-9, miR-125a-5p, and miR-155, which were all identified as being responsible for the regulation of the lipid uptake by macrophages [140]. Furthermore, miR-33, miR-106, miR-122, and miR-144 were shown to control lipid homeostasis, and miR-758 apparently targeted the transcripts that were involved in cholesterol metabolism and fatty acid oxidation [137]. Finally, miR-17-5p, miR-20a, miR-106a, and miR-424 were all shown to regulate monocyte/macrophage differentiation [138].

The expression of miR-125b is related to ATH through its ability to downregulate the expression of podocalyxin (PODXL), an adhesion molecule of endothelial cells [141]. We demonstrated the upregulation of miR-125b in an experimental model of ATH progression and in human ATH plaques [142], and these changes were reversed upon *CD40* silencing [142].

### 5.1. Role of Alu Elements in the Regulation of ANRIL Function

The great interest in *ANRIL* in the context of CAD research arises from this locus harboring a risk allele that is strongly associated with ATH [143]. Recent work has highlighted a link between the regulation of the *ANRIL* function and the presence of *Alu* elements [77,143]. In this work, the authors firstly performed an expression analysis in an effort to characterize the pattern of the *ANRIL* isoforms that were expressed in human peripheral blood mononuclear cells (PBMCs), and in the monocyte cell line, MonoMac. The subsequent analysis of the *ANRIL* expression in CAD patients and control patients demonstrated that the *ANRIL* expression was significantly increased in the samples harbouring the risk allele. The *ANRIL* overexpression had also caused the upregulation of other mRNA transcripts that were related to cell adhesion, growth, and proliferation, an effect that was reversible via the downregulation of *ANRIL* with a specific small interfering RNA (siRNA) [143]. The mechanism through which *ANRIL* was able to trans-regulate the expression of a number of genes required the binding of polycomb repressive complexes 1 and 2 (*PRC1/2*), *CBX7*, *SUZ12*, and others, to *ANRIL*. These proteins were recruited to the promoters of their target genes upon *ANRIL* expression. A bioinformatic analysis of *ANRIL* and of the promoter regions of the *ANRIL*-targeted genes highlighted a common presence of *Alu* elements in both, suggesting that *ANRIL* might bind to chromatin through an *Alu*-mediated interaction, guiding the PRC proteins to *ANRIL*-regulated genes, so as to modify their expression. In this way, the *ANRIL* overexpression could increase the cell proliferation and adhesion, and decrease apoptosis, thus modulating pro-atherogenic cell functions. Contrarily, the *ANRIL* silencing reversed the trans-regulation and normalized the cellular functions [143] (Figure 2A).

The *ANRIL* murine orthologous sequence was encoded in chromosome 4 [144], although the locus was not fully conserved between mice and humans [145]. Interestingly, a murine mutant showing a 70 kb deletion of noncoding DNA in the *ANRIL* locus, which included the risk allele, showed a markedly decreased expression of *Cdkn2a* and *Cdka2b*, as well as an increased proliferation and diminished senescence of primary aortic smooth muscle cells (SMCs) in the culture [144]. These findings strongly supported the hypothesis that the *ANRIL* locus could be implicated in the pathogenesis of the CAD. Nevertheless, the fact that mice did not have bona fide *Alu* elements [146], but rather, they had structurally related B1 elements (see Reference [147] for a recent review), made it difficult to determine the mechanism of action, and to compare it with that of the human *ANRIL*.

### 5.2. Interaction between Alu-RNAs and miRNAs Creates Complex Regulatory Networks

*Alu* elements and miRNAs interact in multiple and complex ways, with the *Alu* elements being a source of miRNAs, which, in turn, target the *Alu* sequences [148]. As previously stated, many of the *Alu* elements that are embedded in the genome have functional RNA Pol III promoters, giving them the ability to be transcribed autonomously and independently of the RNA Pol II transcriptional machinery [18,75]. In a number of cases, these *Alu* promoters had also been seen to prime the RNA-Pol III-dependent (Pol II-independent) transcription of miRNAs [149], and a recent bioinformatic analysis showed that up to 5% of the intronic miRNA genes contained these upstream Pol III-dependent *Alu* regulatory elements [150]. This could explain the discordant expression rates of some intronic miRNAs and their “host” genes. Unfortunately, only a few of these Pol III-dependent miRNAs are currently functionally characterized [149]; however, over 50 miRNAs that were reliant on RNA Pol III for expression were detected within the *Alu* repetitive elements [151].

Another example of *Alu*/miRNA cross-regulation comes from a reported epigenetic therapy of human gastric tumors, which showed that a number of miRNAS were activated via Pol-II-dependent transcription, upon treatment [152]. In this work, the authors showed that the most significant hit (miR-512-5p) was located in close proximity to an *Alu* repeat, which behaved as an RNA Pol II promoter [152]. Similarly, other *Alu* repeats were characterized as Pol II promoters in CpG islands in human genes [153].

On the other hand, there were several reports on the mutual functional relationship between *Alu* elements and miRNAs, resulting in the inactivation of one of these transcripts. Correspondingly, almost 30 human miRNAs were shown to exhibit a short-seed homology with highly conserved *Alu* sequence elements located at the 3′ UTRs of human mRNAs [154], suggesting that these miRNAs could target the mRNA through the *Alu* sequences. Daskalova et al. showed that the majority of the *Alu* sequences that were inserted in the analyzed 3′ UTRs of the human genes carried strong potential target sites for over 50 different miRNAs [155]. Furthermore, in an interesting development, Lehnert et al. showed that the most common miRNA target site coincided with the most conserved sequence of the *Alu* element [156]. More recently, Di Ruocco et al. showed that *Alu*-RNAs were able to induce epithelial–mesenchymal transition (EMT) in colorectal cancer cell lines, by acting as a molecular sponge of miR-566 [157]. Furthermore, miR-15a-3p and miR-302d-3p, which were upregulated during a stress response, were shown to exclusively target the checkpoint DNA exonuclease (RAD1), the G2 and S phase-expressed protein 1 (GTSE1), the nuclear receptor subfamily 2, group C, member 1 protein (NR2C1), the FK506-binding protein 9 (FKBP9), and the ubiquitin-conjugating enzyme E2 (UBE2I), within the *Alu* elements [158]. Additionally, miR-661 was found to target the MDM2 proto-oncogene (*Mdm2*) and *Mdm4*, resulting in their downregulation, with a subsequent increase in p53 activity; the inhibition of cell cycle progression in p53-proficient cells was also shown to occur within the *Alu* elements [159]. In this context, and although much more work is required on the interaction between the *Alu*-RNAs and miRNAs, we hypothesize that individual *Alu*-RNAs or mRNA-embedded *Alu* elements could have an impact on ATH progression, by behaving as molecular sponges for specific miRNAs that are involved in disease development (Figure 2B).

### 5.3. Alu Elements Are Common Binding Sites for Transcription Factors, Such as NF-κB, and May Impact Gene Expression of the Inflammatory Response

NF-κB proteins are critical regulators of the immune response, with a substantiated role in ATH progression in animal models of ASVD [160], and in human ASVD [161,162] patients. Activated NF-κB was localized in vascular endothelial cells (VEC), smooth muscle cells (SMCs), and lymphocytes in the vasa vasorum of the abdominal aortas, with atherosclerotic plaques that were isolated from deceased patients [162]. NF-κB can be activated through two different pathways, one canonical and the other non-canonical. In the canonical pathway, the NF-κB nuclear factors (RelA or p65, RelB, c-Rel, p50, and p52) remained inactive in the cytoplasm by interacting with the inhibitor of kappa B (IκB) proteins. The triggering of various receptors, including pattern recognition receptors (PRRs), tumor necrosis factor receptors (TNFRs), T-cell receptors (TCRs), B-cell receptors (BCR), etc., activated the IκB kinases (IKKs), which resulted in IκB phosphorylation, and subsequent proteasomal degradation, thus facilitating the nuclear translocation of NF-κB RelA/p50, and the subsequent activation of the NF-κB downstream target genes [163]. In the alternative, non-canonical NF-κB pathway, NF-κB inducing kinase (NIK) activated IKKκ, which phosphorylated and processed p100 to p52, inducing the formation of a transcriptionally active RelB/p52 complex [163]. On this topic, we recently described the upregulation of the activator *IKKκ* gene and the downregulation of the *IKBα* inhibitor gene during disease progression, in the experimental ApoE^−/−^ model of ATH [142], suggesting the role of canonical NF-κB activation in ATH progression.

NF-κB nuclear factors (RelA, RelB, and c-Rel) had bound to the consensus κB site (5′-GGGRNYYYCC-3′) in the promoters or enhancers of the target genes [163]. NF-κB was also shown to bind to many non-consensus sites [164], of which nearly 10% were detected in *Alu* repetitive elements, termed *Alu*-κB elements [165]. Although only a few of them were directly correlated with changes in the expression of associated genes, it was suggested that these *Alu*-κB elements could perform other cell type-specific functions, such as sequestering transcriptionally inert NF-κB molecules, which would allow competent factors to activate target genes, but prevent the excessive targeting and superactivation of promoters [165]. These data suggest that *Alu* elements, combined with other nearby cis-acting elements, might play an important role in expanding the repertoire of the NF-κB binding sites, allowing the engagement of new genes into NF-κB-dependent regulatory networks (Figure 2C). Furthermore, the genome-wide chromatin immunoprecipitation sequencing (ChIP-seq) in various individuals and cell lines demonstrated that the NF-κB binding sites are polymorphic, and can differ by over 7.5% among individuals. Most of these differences were as a result of SNPs in intergenic regions, and were correlated with the differences in gene expression, indicating that the polymorphic variation in binding sites could have functional consequences [166]. Although no data are available on the extent to which the binding variation occurs in the *Alu* elements, it could be expected that significant differences in transcription-factor binding sites, as well as in the gene expression between individuals, are as a result of polymorphic *Alu* repeats.

### 5.4. A Polymorphic Alu Insertion Controls the Renin-Angiotensin System

It is known that elevated levels of angiotensin II (Ang II) contribute to vascular disease, and that the kidney plays a critical role in the regulation of the renin–angiotensin–aldosterone system (RAAS) [167]. Kidney renin is released into the blood, where it cleaves circulating angiotensinogen into angiotensin I, which is subsequently transformed into angiotensin II by the angiotensin-converting enzyme (*ACE*) that is produced in the vascular endothelium. Ang II may accelerate ATH progression through the activation of factors, such as NF-κB, adhesion molecules, transforming growth factor (TGF)-β, or endothelin-1, thereby inducing vascular growth, cell migration, and inflammation [168]. In addition, Ang II is a potent stimulus for pro-oxidant enzymes, leading to an increase in the production of reactive oxygen species (ROS), and consequently, to increased oxidative stress. On the contrary, blocking RAAS has demonstrated beneficial effects for the treatment of cardiovascular and renal disease [169].

*Alu* elements are involved in the regulation of RAAS, and consequently, in the progress of renal [170] and cardiovascular diseases [171], by virtue of a polymeric *Alu* insertion in intron 16 of the *ACE* gene, giving rise to two different alleles, namely, the “insertion allele” (I allele) and the “deletion allele” (D allele). The insertion allele of the *Alu* element (I allele) in the *ACE* gene resulted in an open reading frame (ORF) shift, resulting in the premature termination of the *ACE* protein, and the generation of a protein with a single active site in the N-terminal domain [69]. Homozygous D/D individuals have plasma *ACE* levels that are about twice as high as those of the homozygous I/I individuals [172], and also demonstrate diminished levels of tissue *ACE* [173]. Surprisingly, the *Alu* repeat was found to also upregulate the transcriptional activity of the *ACE* promoter [174]. However, associated studies on the I/D polymorphisms of the *ACE* gene and cardiovascular outcomes are still controversial because of the lack of powered studies and the existence of interactions with other genes or environmental factors [175]. Furthermore, although a number of recent meta-analyses have highlighted *ACE* I/D risk associations with hypertrophic cardiomyopathy [176], ischemic stroke [177], and increased CKD [178], ethnicity still remains a strong confounder. This is evidenced by the population-restricted associations that are described for the *ACE* I/D polymorphism, with susceptibility to abdominal aortic aneurysms in European populations, but not in Asian populations [179]. On the other hand, it was strongly associated with ischemic stroke in Asian populations, but had borderline statistical significance for Caucasians [180].

## 6. Concluding Remarks

Although great efforts were made to explore the landscape of the molecular alterations underlying the development of ATH, and despite the wealth of knowledge that has been generated, we still have a limited vision of most of the mechanisms that are involved in ATH development, as well as their interactions or their mutual interferences. Clearly, new approaches for ATH research are required in order to integrate new tiers of information and new regulatory layers. On this basis, the chaos theory and the study of nonlinear dynamic systems offer new conceptual approaches, and provide insight to better understand highly complex problems [181]. Chaotic systems, which can be defined as deterministic but not predictable, are characterized by their exquisite sensitivity to their initial conditions, and develop by following the trajectories of strange attractors of a fractal nature [182]. A number of authors have proposed a chaotic component in the development of atherosclerosis [183,184,185], a hypothesis that was subsequently confirmed by the work of Xiao et al., who used a chaos theory-derived nonlinear prediction method to highlight deterministic (non-random) structures in the repetitive *Alu* elements. These structures were as a result of their dimeric composition, a likely basis for nonlinear regulatory behaviour [16]. These results were further backed by Moreno et al., who used a multifractal approach to study the human genome, showing that a multifractal pattern was strongly correlated with the presence of repeated elements of the *Alu* family [43]. These works made conceptual links between disease (ATH) and the overall structure of the human genome; they also focused our attention on the functional involvement of *Alu* repeated elements in ATH progression. In conclusion, the advent of the genomic revolution highlighted the involvement of noncoding DNAs and RNAs in human diseases. Here, we reviewed the available data on the role of two such noncoding nucleic acids—the lncRNA *ANRIL*, and the family of *Alu* repeated elements—on ATH onset and progression. Although much research remains to be done on these (and other) noncoding elements, it is becoming clear that the natural history of human disease is no longer simply a question of proteins and coding genomic regions. Noncoding regions and long-range sequence correlations do, in fact, have an important role in disease development.

## Abbreviations

asRNA	antisense transcripts from coding regions
ASVD	arteriosclerotic vascular disease
ATH	Atherosclerosis
CAC	coronary artery calcification
CAD	coronary artery disease
circRNA	circular noncoding RNA
eQTL	expression quantitative trait locus
ECM	extracellular matrix
EMT	epithelial–mesenchymal transition
FH	familial hypercholesterolemia
GWAS	genome-wide association studies
HIV	human immunodeficiency virus
LINE	long interspersed nuclear element
ncRNA	noncoding RNA
lncRNA	long noncoding RNA
lincRNA	long intergenic noncoding RNA
MACE	major adverse cardiovascular event
miRNA	microRNA
piRNA	piwi-interacting RNA
PRC	polycomb repressive complex
PRR	pattern recognition receptor
RAAS	renin–angiotensin–aldosterone system
ROS	reactive oxygen species
SMC	smooth muscle cells
SNP	single-nucleotide polymorphism
SINE	short interspersed nuclear element
SRP	signal recognition particle
TSS	transcription start site
UTR	untranslated region
VEC	vascular endothelial cell
Ang II	angiotensin II
*ACE*	angiotensin-converting enzyme
*ANRIL*/*CDKN2B-AS1*	antisense noncoding RNA in the *INK4* locus, *CDKN2B* antisense 1
BCR	B-cell receptor
*CBX7*	chromobox protein homolog 7
CDK	cyclin-dependent kinase
FKBP9	FK506 binding protein 9
GTSE1	G2 and S phase-expressed protein 1
HDL	high-density lipoprotein
HGH	human growth hormone
IKK	IκB kinase
INK	inhibitor of CDK4
LDL	low-density lipoprotein
*MDM2*	*MDM2* proto-oncogene
*MDM4*	*MDM4* p53 regulator
MMP-9	matrix metalloproteinase 9
NEMO	NF-κB essential modulator
NIK	NF-κB-inducing kinase
*NF1*	neurofibromatosis 1 gene
NF-κB	activated nuclear factor kappa B
*NR2C1*	nuclear receptor subfamily 2 group C member 1
oxLDL	oxidized LDL
PODXL	podocalyxin
*RAD1*	checkpoint DNA exonuclease
RANK	receptor activator of NF-κB
STAT1	signal transducer and activator of transcription 1
SUZ12	suppressor of zest 12 (subunit of polycomb repressive complex 2)
TCR	T-cell receptor
TNFR	tumor necrosis factor receptor
UBE2I	ubiquitin-conjugating enzyme E2
VEGF	vascular endothelial growth factor
